# Evaluating the utility of the female-specific mitochondrial *f-orf* gene for population genetic, phylogeographic and systematic studies in freshwater mussels (Bivalvia: Unionida)

**DOI:** 10.7717/peerj.5007

**Published:** 2018-06-13

**Authors:** Brent M. Robicheau, Emily E. Chase, Walter R. Hoeh, John L. Harris, Donald T. Stewart, Sophie Breton

**Affiliations:** 1 Department of Biology, Acadia University, Wolfville, Canada; 2 Department of Biological Sciences, Kent State University, Kent, United States of America; 3 Department of Biological Sciences, Arkansas State University, Jonesboro, United States of America; 4 Department of Biological Sciences, University of Montreal, Montreal, Canada; 5 Current affiliation: Department of Biology, Life Science Centre, Dalhousie University, Halifax, Canada

**Keywords:** Unionida, Freshwater mussels, Mitochondrial DNA, Bivalvia, DNA barcode, Doubly uniparental inheritance of mtDNA, Molecular markers

## Abstract

Freshwater mussels (order: Unionida) represent one of the most critically imperilled groups of animals; consequently, there exists a need to establish a variety of molecular markers for population genetics and systematic studies in this group. Recently, two novel mitochondrial protein-coding genes were described in unionoids with doubly uniparental inheritance of mtDNA. These genes are the *f-orf* in female-transmitted mtDNA and the *m-orf* in male-transmitted mtDNA. In this study, whole F-type mitochondrial genome sequences of two morphologically similar *Lampsilis* spp. were compared to identify the most divergent protein-coding regions, including the *f-orf* gene, and evaluate its utility for population genetic and phylogeographic studies in the subfamily Ambleminae. We also tested whether the *f-orf* gene is phylogenetically informative at the species level. Our preliminary results indicated that the *f-orf* gene could represent a viable molecular marker for population- and species-level studies in freshwater mussels.

## Introduction

Freshwater mussels (Bivalvia: Unionida) occur globally, except in Antarctica, with more than 800 estimated species (Bogan & Roe, 2008). Despite high diversity, many species are critically imperilled (Regnier, Fontaine & Bouchet, 2009; Lopes-Lima et al., 2017). Approximately 70% of the ∼300 North American species are endangered at some level (Lopes-Lima et al., 2017). Freshwater mussels are well recognized for their water filtration capabilities, and for the production of obligate parasitic larvae that metamorphose on fish hosts (Regnier, Fontaine & Bouchet, 2009; Lopes-Lima et al., 2017). They also possess an unusual system of mitochondrial transmission called doubly uniparental inheritance (DUI), a characteristic shared with various other bivalves (Gusman et al., 2016). DUI is the only exception to the strictly maternal inheritance of mitochondrial DNA (mtDNA) in animals and is characterized by having two types of mtDNA in males (the male-transmitted or M-type mtDNA in germline cells and the female-transmitted or F-type mtDNA in soma), and usually one type (the F-type) in females (Breton et al., 2007). DNA divergence between M and F mtDNAs within a single male freshwater mussel can reach >40% (Doucet-Beaupré et al., 2010). Moreover, each sex-associated mtDNA contains a novel protein-coding gene in addition to the 13 typical genes involved in ATP production (*m-orf* in M-type and *f-orf* in F-type mtDNA; Breton et al., 2009; Breton, Stewart & Hoeh, 2010; Milani et al., 2013). These genes are among the fastest evolving mt genes in freshwater mussels (Breton et al., 2011a; Breton et al., 2011b; Mitchell et al., 2016). They also have hypothesized roles in the maintenance of DUI and sex determination in bivalves (Breton et al., 2011a; Breton et al., 2011b), with recent *in silico* analyses supporting such hypotheses (Mitchell et al., 2016).

Molecular techniques are commonly used to study freshwater mussels (Mulvey et al., 1997; Krebs, 2004; Campbell et al., 2008) since shell morphology alone is often inadequate to define populations, species or subfamilies. Environmental conditions can affect shell developmental patterns and obfuscate taxonomic identification (Bogan & Roe, 2008). Recent divergence (and retention of ancestral morphological characteristics) and hybridization phenomena also make shell characters only partially efficient in discriminating certain populations or lineages (Hoeh et al., 1995; Cyr et al., 2007). For example, significant genetic differences have been discovered in *Utterbackia* populations with little to no apparent differences in shell morphology (Hoeh et al., 1995). Several studies have used both F- and M-type mtDNA sequences. For example, fragments of the 16S rRNA and cytochrome c oxidase subunit I (*cox1*) genes obtained with the universal primers 16Sar-5 and 16Sbr-3 (Palumbi et al., 1991) and HCO2198 and LCO1490 (Folmer et al., 1994), respectively (or with modified versions of the latter two (Walker et al., 2006)), have been used to answer systematic, phylogenetic (Krebs, 2004; Krebs et al., 2013; Doucet-Beaupré et al., 2012) and phylogeographical (Mioduchowska et al., 2016) questions about freshwater mussels. Since the M-type mt genomes typically evolve faster than their F-type counterpart in freshwater mussels (Krebs, 2004; Gusman et al., 2016), relatively older (i.e., species- or family-level) divergences may be tracked more accurately with analyses of the more slowly evolving F-type mtDNA, while analyses of relatively recent (e.g., population-level) divergences may be facilitated by analyses of the faster evolving M-type mtDNA. For example, only the faster evolving male form of the 16S rRNA gene provided strong evidence of geographical isolation among *Pyganodon grandis* populations from the southern region of the Lake Erie watershed (Ohio, USA) (Krebs, 2004). However, because the male mtDNA is restricted to the testes, this requires identification of males, and this is impossible with juvenile specimens or with larvae (glochidia). Moreover, the precarious situation of several freshwater mussel species sometimes require non-destructive sampling of animals (e.g., using mantle snips and thus with no access to the M-type mtDNA), which are then returned to the river bottom (Inoue et al., 2013).

To explore species boundaries, evolutionary relationships and geographic distribution of freshwater mussel species, researchers also tried other protein-coding loci of the F-type mtDNA such as cytochrome c oxidase subunit II (*cox2*; Doucet-Beaupré et al., 2012) and NADH dehydrogenase subunit 1 (*nad1*; Campbell et al., 2005). For example, Campbell et al. (2005) used *nad1* together with *cox1* and 16S rRNA to study the phylogenetic diversity of the subfamily Ambleminae, but their data could not resolve all the tribes (Amblemini, Lampsilini, Pleurobemini, Quadrulini) as monophyletic assemblages. The same three gene fragments were also found to be poor at resolving recent relationships (intrageneric level) by other researchers (e.g., Sommer, 2007; McCartney et al., 2016).

Recently, Wares (2014) used a straightforward approach and compared whole mitochondrial genome sequences of recently-diverged taxa to identify the most divergent protein-coding region and verify its utility for population genetics (see also Shearer & Coffroth, 2008) and systematic studies in scleractinian corals. Although its results suggested that this region alone (cytochrome b, *cytb*) was unlikely to improve researchers’ ability to separate coral taxa using DNA sequence-based methods, the proposed pipeline, i.e., to find the most divergent region and to analyze its divergence across available GenBank data, could certainly be adopted to find another useful F-type mitochondrial region for population genetics and systematic studies in freshwater mussels.

Following Wares’ pipeline, we focused on Ambleminae, an important freshwater mussel subfamily with several species listed as threatened or endangered (IUCN, 2015), and searched for another useful region in the F-type mtDNA to explore phylogenetic diversity and phylogeographic or population genetic structure in this taxa. We compared F mt genomes between two putative species (i.e., two species that are difficult to tell apart morphologically), the Arkansas Fatmucket, *Lampsilis powellii* (I. Lea, 1852) and the Fatmucket, *Lampsilis siliquoidea* (Barnes, 1823) (Harris et al., 2004; Harris et al., 2010; Krebs et al., 2013), and identify highly divergent protein-coding regions such as the *f-orf* gene. We then analyzed sequence divergence in this region (and test whether it is phylogenetically informative) across available amblemine data as a first step to see if it could represent a viable molecular marker for population- and species-level studies in freshwater mussels.

## Materials and Methods

*Lampsilis* mussels were collected from two major river drainages in the state of Arkansas: Ouachita River drainage - Ouachita River (Polk County = isolate H2610), and Red River drainage - Mountain Fork Little River (Polk County = isolate H2655). Specimens were obtained under permit, including Arkansas Game and Fish Commission Scientific Collection Permits Nos. 022220078 and 062220101, and Federal Fish and Wildlife Permit No. TE079883-2 issued to JL Harris. Samples were identified as *Lampsilis siliquoidea* or *L. powellii* according to Harris et al. (2004) and Harris et al. (2010), i.e., based on external shell morphology (color rays absent, pit rays present, nacre color matte yellow to tan = *L. powellii*; color rays present, pit rays absent, nacre color shiny yellow to tan = *L. siliquoidea*). Each individual was sexed through microscopic examination of gonad tissues. Total DNA was extracted from female mantles to obtain the female-transmitted mtDNA using a QIAGEN DNeasy animal kit following the manufacturer’s protocol. Complete mtDNAs were PCR amplified, according to the method of Breton et al. (2011b) using primers listed in Table 1. Purified products were sequenced using FLX sequencing (McGill University and Genome Quebec Innovation Centre).

**Table 1:** Primers pairs used in the amplification of the entire F genomes.

Mitotype Region (Amplicon size)	Primer name	Primer sequence (5′ to 3′)
**F genome**		
*cox2 –rrnL* (∼11 kb)	*UNIOCOII.2^a^ Ambl16SFor^c^	CAGTGGTATTGGAGGTATGAGTA CTGGGTTTGCGACCTCGATGTTGGCTTAGGGAAA
*cox1 –rrnL* (∼5.5 kb)	*HCO-700y2^b^ Ambl16SRev^c^	TCAGGGTGACCAAAAAAYCA TTTCCCTAAGCCAACATCGAGGTCGCAAACCCAG

**Notes.**

For primer names: Ambl and *, Amblemine-specific primers.

a From Curole & Kocher (2002).

b From Walker et al. (2006).

c See Breton et al. (2011b).

Sequences were assembled with MacVector v10.0 (Rastogi, 1999), annotated using MITOS (Bernt et al., 2013), and compared to published freshwater mussel mtDNAs. Further assessment of tRNA genes used *tRNAScan-SE* v1.21 (Lowe & Eddy, 1997). MUSCLE (Edgar, 2004) was used within Geneious v10.0.9 (Kearse et al., 2012) to align complete mtDNAs. Nucleotide divergence K(JC) across F-to-F mt genomes was determined with DnaSP v5 (sliding-window = 500 bp; step size = 25 bp) (Librado & Rozas, 2009).

DNA alignments of individual genes were produced via MUSCLE in MEGA7 (Kumar, Stecher & Tamura, 2016). MEGA7 was used to: determine *p*-distances and *dN*/*dS* values (*dN* = nonsynonymous substitutions/nonsynonymous sites; *dS* = synonymous substitutions/synonymous sites), calculate *Z*-tests, and generate trees. The best substitution model for each gene was chosen via a model selection test in MEGA7, alignments were manually trimmed to start/end positions without gaps, and Maximum-likelihood (ML) trees were generated with 500 bootstrap replicates (complete deletion was used to account for gaps in ML trees). Bayesian inference trees were produced via BEAST v2.4.6 (Drummond et al., 2012), using a Hasegawa-Kishino-Yano model for *f-orf* and a Tamura-Nei evolutionary model for *cox1* (based on BEAST modeltest results), a Yule speciation process, and 80 million Markov chain Monte Carlos steps (sampling every 1000 steps). A 10% burn in was used and resulting trees were compiled into the highest probability topology using TreeAnnotator v1.4 (Rambaut & Drummond, 2002). Graphs were produced using ggplot2 within R Team (2015). Specimens used in our phylogenetic analyses and in analysis of *f-orf* and *cox1* sequences variability are described in Table 2. Nomenclature followed Williams et al. (2017). Mitochondrial genomes were deposited in GenBank (accession nos. MF326971 and MF326973).

**Table 2:** *Cox1* and *f-orf* sequences used in Ambleminae phylogenies and in analysis of *f-orf* and *cox1* sequences variability.

Species	*cox1*		*f-orf*	
	Accession	Reference	Accession	Reference
*Ellipsaria lineolata*	AY654994	Campbell et al. (2005)	HM849378^a^	Breton et al. (2011b)
	GU085285^a^	Boyer et al. (2011)	–	–
	HM849071	Breton et al. (2011b)	–	–
*Fusconaia flava*	DQ298537^a^	Burdick & White (2007)	HM849380	Breton et al. (2011b)
	DQ298538	Burdick & White (2007)	HM849381^a^	Breton et al. (2011b)
	EF033261	Chapman et al. (2008)	HM849382	Breton et al. (2011b)
	HM849073	Breton et al. (2011b)	–	–
*Lampsilis ornata*	AF385112	Roe, Hartfield & Lydeard (2001)	AY365193^a^	Serb & Lydeard (2003)
	AY365193^a^	Serb & Lydeard (2003)	–	–
*Lampsilis powellii*	HM849075^a^	Breton et al. (2011b)	MF326971^a^	This study
	–	–	HM849384	Breton et al. (2011b)
*Lampsilis siliquoidea*	HM849076	Breton et al. (2011b)	MF326973^*^	This study
	–	–	HM849385	Breton et al. (2011b)
*Lemiox rimosus*	AY655002^a^	Campbell et al. (2005)	–	–
	EF033256	Chapman et al. (2008)	–	–
	HM849093	Breton et al. (2011b)	–	–
*Megalonaias nervosa*	AY655007^a^	Breton et al. (2011b)	HM849404^a^	Breton et al. (2011b)
*Potamilus metnecktayi*	HM849099^a^	Breton et al. (2011b)	HM849405^a^	Breton et al. (2011b)
	–	–	HM849406	Breton et al. (2011b)
*Quadrula quadrula*	FJ809750^a^	Breton et al. (2009)	FJ809750^a^	Breton et al. (2009)
	KX853888 –KX853982	Mathias et al. (2018)	–	–
*Reginaia ebenus*	AY654999	Unpublished	HM849379^a^	Breton et al. (2011b)
	HM849072	Breton et al. (2011b)	–	–
	KF035133^a^	Inoue et al. (2013)	–	–
*Cyclonaias houstonensis*	KT285649^a^	Pfeiffer et al. (2016)	HM849440^a^	Breton et al. (2011b)
	–	–	HM849441	Breton et al. (2011b)
*Cyclonaias tuberculata*	GU085284^a^	Boyer et al. (2011)	HM849376^a^	Breton et al. (2011b)
	HM849069, HM849070	Breton et al. (2011b)	HM849377	Breton et al. (2011b)
*Toxolasma lividum*	AF231756^a^	Bogan & Hoeh (2000)	HM849451, HM849452, HM849453, HM849454, HM849455	Breton et al. (2011b)
	JF326436	Campbell & Lydeard (2012)	HM849456^a^	Breton et al. (2011b)
*Toxolasma sp. aff. paulum 1*	HM849131^a^	Breton et al. (2011b)	HM849458	Breton et al. (2011b)
	HM849133	Breton et al. (2011b)	HM849459, HM849460, HM849461, HM849462, HM849463,	Breton et al. (2011b)
	–	–	HM849464^a^	Breton et al. (2011b)
*Toxolasma sp. aff. paulum 2*	HM849129^a^	Breton et al. (2011b)	HM849465, HM849466, HM849467, HM849468, HM849469, HM849470, HM849471, HM849472, HM849473	Breton et al. (2011b)
	HM849130	Breton et al. (2011b)	HM849474^a^	Breton et al. (2011b)
	HM849132	Breton et al. (2011b)	HM849475	Breton et al. (2011b)
*Toxolasma pullus*	–	–	MF326970^a^	This study
*Toxolasma texasiense*	AY655023^a^	Campbell et al. (2005)	HM849476	Breton et al. (2011b)
	–	–	HM849477^a^	Breton et al. (2011b)
*Truncilla macrodon*	HM849165^a^	Breton et al. (2011b)	HM849478^a^	Breton et al. (2011b)
	KT285658	Pfeiffer et al. (2016)		
*Venustaconcha ellipsiformis*	EF033260^a^	Chapman et al. (2008)	FJ809753	Breton et al. (2009)
	–	–	HM849529^a^	Breton et al. (2011b)
	–	–	HM849530	Breton et al. (2011b)
*Villosa iris*	HM849199^a^	Breton et al. (2011b)	HM849531	Breton et al. (2011b)
	HM849200, HM849201	Breton et al. (2011b)	HM849532^a^	Breton et al. (2011b)
	–	–	HM849533	Breton et al. (2011b)

**Notes.**

*Cox1* sizes range from 339 to 1,541 bp. *F-orf* sizes range from 240 to 410 bp. Note that for trees sequences were trimmed to a common start and end position in alignments.

a Sequences that were specifically used in Ambleminae phylogenies.

## Results

Mitochondrial genome sizes (*F* = 16,043 and 16,990 bp for *L. powelli* and *L. siliquoidea*, respectively), gene order and compositions are consistent with those of other freshwater mussels (Doucet-Beaupré et al., 2010). We detected one large, potentially species-specific indel: a 51 bp indel in the *cox2/nad3* spacer region between the two F genomes.

**Figure 1:**
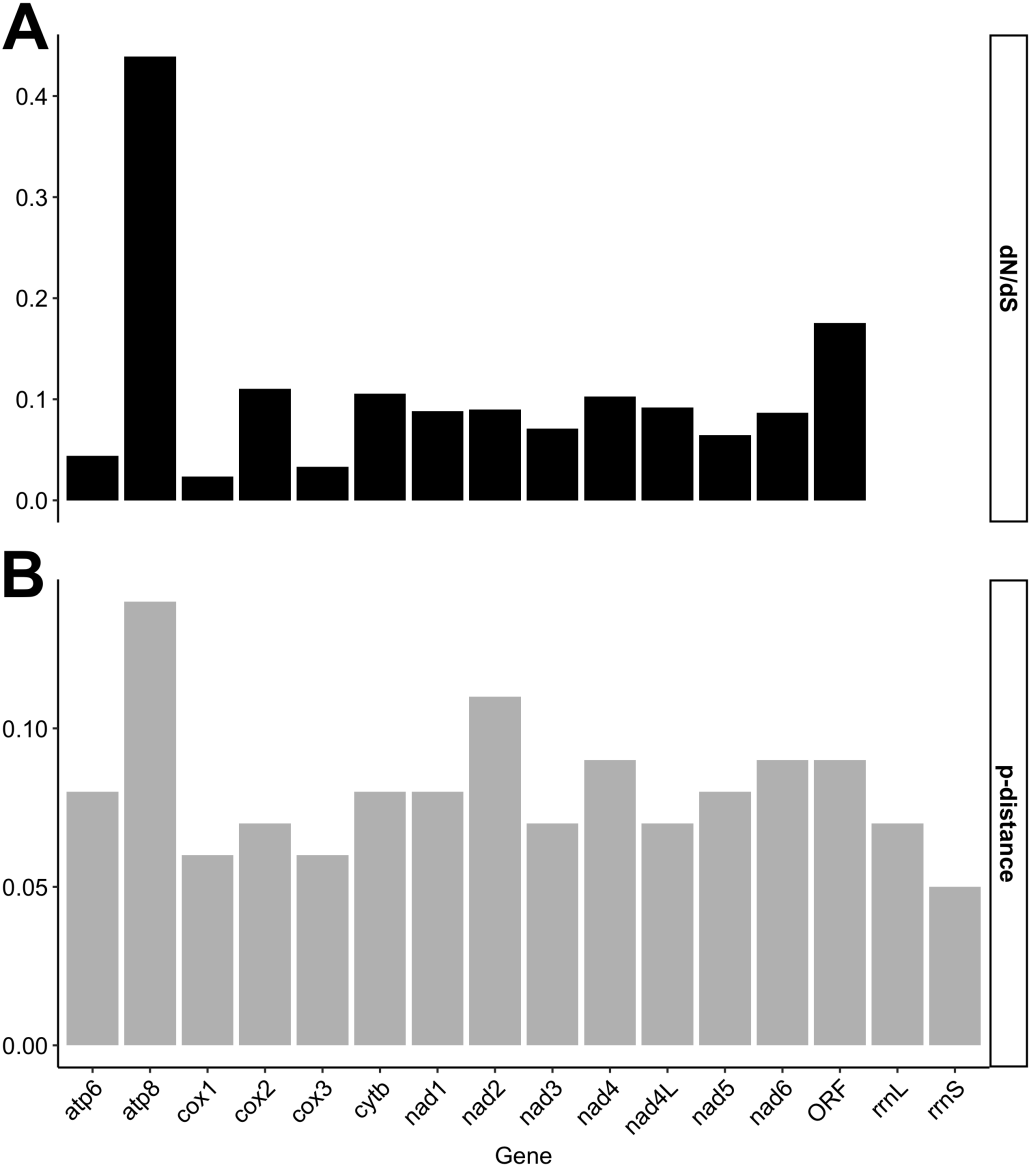
Nucleotide distances calculated for individual mitochondrial gene comparisons. (A) *dN*/*dS* ratios/scores; (B) *p*-distance scores.

Individual gene *p*-distances and *dN*/*dS* ratios are given in Fig. 1. Our results show, consistent with the degree of nucleotide divergence across mt genes in F genomes (Fig. S1), that the *f-orf* gene is among the least conserved genes in F genomes (Fig. 1). Only *atp8* has a higher *dN*/*dS* ratio (>0.4) than *f-orf*, although a *Z*-test for selection indicated that the probability of rejecting the null hypothesis of *dN* = *dS* (neutrality) for this gene was 0.092.

**Figure 2:**
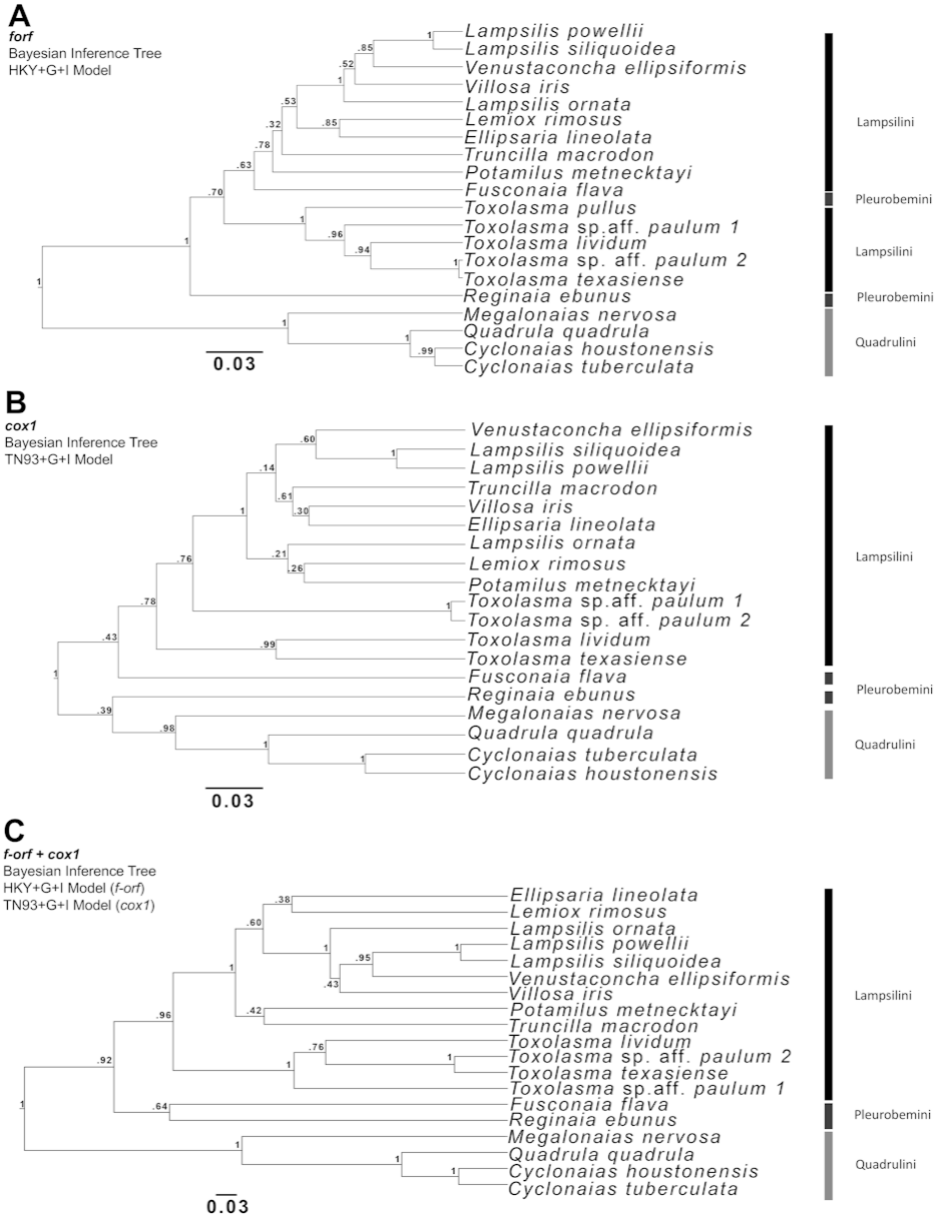
Bayesian inference (BI) trees produced in BEAUti and BEAST 2.4.6 for (A) *f-orf,* (B) *cox1*, and (C) concatenated *f-orf* and *cox1* sequences also used in maximum likelihood trees of Fig. S2. (A) was produced using a Hasegawa-Kishino-Yano model, (B) a Tamura-Nei model and (C) both these models of nucleotide substitution. Sequences used in BI trees refer to those listed and starred in Table 2.

Phylogenetic analyses focused on the *f-orf* gene because several *f-orf* sequences are available in GenBank compare to *atp8*. Three Bayesian phylogenetic trees were built for Ambleminae using: the *f-orf* gene (Fig. 2A), the standard animal mitochondrial *cox1* DNA barcode (Hebert, Ratnasingham & De Waard, 2003; Fig. 2B), and both genes concatenated (Fig. 2C). Corresponding ML analyses are in Fig. S2. The *f-orf* sequences led to better bootstrap values than *cox1*. Moreover, members of *Toxolasma* were grouped as a single clade only in the *f-orf* containing trees. Similar results were obtained with ML analyses. BI and ML trees with the highest bootstrap values were obtained using both *f-orf* and *cox1* together.

**Figure 3:**
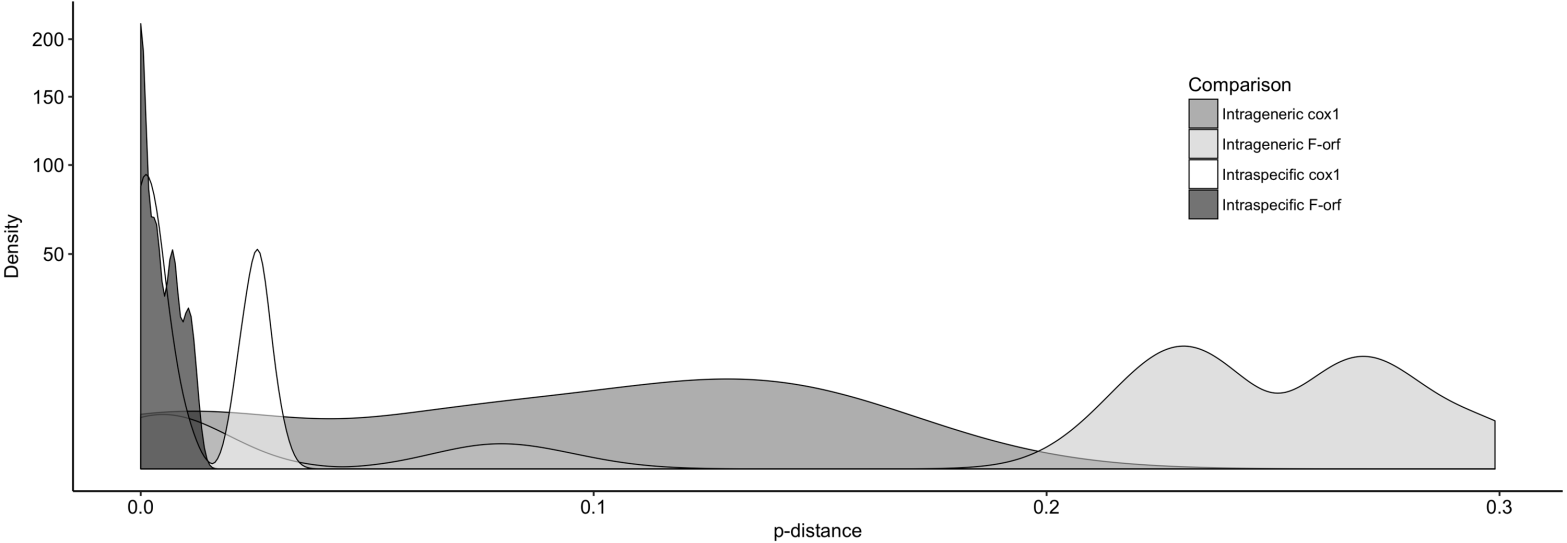
The distribution of pair-wise *p*-distance scores for *f-orf* and *cox1* genes within Ambleminae. The taxa compared are listed in Table 2. Intrageneric comparisons exclude intraspecific comparisons.

We further examined Ambleminae *f-orf* variability, again using *cox1* for comparison (Fig. 3). For the *f-orf* gene*,* intraspecific comparisons have lower variability and smaller *p*-distances (range = 0.000–0.011) versus *cox1* (range = 0.000–0.031), and intrageneric comparisons for the *f-orf* gene have overall greater *p*-distances.

## Discussion

Our research objective was to provide guidance towards the identification of another useful region in the F-type mtDNA to explore phylogenetic diversity and phylogeographic or population genetic structure in freshwater mussels. As pointed out by Wares (2014), finding gene regions that provide sufficient information, above and beyond the variation found within a population, might be challenging and we agree with him that it may be more optimal to first explore available genomic data (mitochondrial or whole mtDNA) rather than only use available primer regions or the same gene region that has proven useful in other animal species. In this study, we show that *f-orf*, and also *atp8*, have high divergence between morphologically similar members of *Lampsilis*. Although *atp8* had the highest *dN/dS* ratios, the pattern was consistent with neutrality. This gene has historically been either missing and/or found to be the least conserved in bivalve mtDNAs (Breton, Stewart & Hoeh, 2010). Therefore, it is not surprising that it evolves in a different manner than all other mt genes.

Based on divergence data, *f-orf* and *atp8* could thus represent valuable molecular markers in the context of population genetic studies in Ambleminae. Such genes, and in particular *f-orf*, could also potentially help test the hypothetical involvement of mitochondria and their genomes in establishing reproductive barriers and speciation events (Gershoni, Templeton & Mishmar, 2009). The general research trend has shown the involvement of sex-linked genes in reproductive isolation (Qvarnström & Bailey, 2009), therefore, demonstrating the participation of F- and M-ORF proteins in sex determination, as predicted by Breton et al. (2011b), would particularly corroborate the potential of mitochondrial genetic speciation mechanisms. There were not enough *atp8* sequences (or whole mitochondrial genome sequences) available in GenBank for Ambleminae to see if they could be more informative for population genetic or species delineation studies, however, enough *f-orf* sequences were available to achieve the brief goal of our study.

At the species level, an optimal marker should have a fairly high level of sequence variability, but at the same time it should be sufficiently conserved to reduce phylogenetic noise. Analyses have suggested the systematic usefulness of the *f-orf* gene, while *atp8* appears too noisy. We proceeded with phylogenetic analyses using *f-orf* compared to *cox1* to evaluate the systematic utility of this gene. The *f-orf* BI phylogeny, like *cox1,* was able to distinguish *L. siliquoidea* from *L. powellii* with high bootstrap support. Overall, our data suggest that both genes are somewhat limited in fully resolving Ambleminae phylogenies on their own, which is illustrated by each tree having relatively low bootstrap values. However, in combination, *f-orf*+*cox1* produced a phylogeny with higher bootstraps, indicating their value for systematic studies.

We further determined *p*-distances in *f-orf* and *cox1* for more sequences within the same lineages. These preliminary data suggest that at the population level, the *f-orf* gene displays relatively fewer nucleotide differences within species, while within genera, there are a relatively larger number of differences (compared to *cox1*). This point is exemplified by *f-orf*s typically having >20% sequence differences between species of the same genus (with the exception of the anomalous *Toxolasma* species pair that had a *p*-distance of <0.05).

## Conclusion

This preliminary study indicates that the f-*orf* gene in freshwater mussels could represent a viable molecular marker for population- and species-level studies. This is based on: (1) the *f-orf* gene experiencing a high degree of relaxed purifying selection; (2) the *f-orf* gene, especially when used in combination with *cox1* (and it remains to be seen whether it is the case with other genes), can be phylogenetically informative, and (3) our detection of generally low within-species variability for the *f-orf*, and relatively high between-species variability for most closely related taxa.

##  Supplemental Information

10.7717/peerj.5007/supp-1 Figure S1 Sliding window analysis of nucleotide divergence between F-versus-F genomes Analysis was conducted using a window size of 500 bp and 25 bp jumps. JC, Jukes Cantor correction. Light grey blocks represent protein-coding OXPHOS and ribosomal RNA gene regions, while the novel *f-orf* gene is highlighted dark grey. Dashed lines indicate the midpoint of genes.

10.7717/peerj.5007/supp-2 Figure S2 Maximum-likelihood Ambleminae trees for *f-orf* (A), *cox1* (B), and concatenated *f-orf* +*cox1* sequences (C) Samples/sequences used are described in Table 2. Substitution models selected (Hasegawa-Kishino-Yano (HKY) +G for *f-orf,* Tamura-Nei (TN93) +G +I for *cox1*, and TN93 +G for *f-orf* +*cox1*) were based on running alignments through MEGA’s model selection analysis. The best models for individual gene alignments had the lowest BIC scores for each gene. The top most similar model between the two individual model tests was chosen as the best model for the concatenated gene tree (again based on lowest BIC scores). Bootstrap percentage values based on 500 replicates are shown to the left of nodes.

10.7717/peerj.5007/supp-3 Supplemental Information 1 Mitochondrial genome sequences Lampsilis spp. F and M mtDNAs.

## Additional Information and Declarations

### Field Study Permissions

The following information was supplied relating to field study approvals (i.e., approving body and any reference numbers): Specimens were obtained under permit, including Arkansas Game and Fish Commission Scientific Collection Permits Nos. 022220078 and 062220101, and Federal Fish and Wildlife Permit No. TE079883-2 issued to JL Harris.

### Data Availability

The following information was supplied regarding data availability: Mitochondrial genomes were deposited in GenBank (accession nos. MF326971 and MF326973).

## References

[ref-1] Bernt M, Donath A, Jühling F, Externbrink F, Florentz C, Fritzsch G, Pütz J, Middendorf M, Stadler PF (2013). MITOS: improved de novo metazoan mitochondrial genome annotation. Molecular Phylogenetics and Evolution.

[ref-2] Bogan AE, Hoeh WR, Harper EM, Taylor JD, Crane JA (2000). On becoming cemented: evolutionary relationships among the genera in the freshwater bivalve family Etheriidae (Bivalvia: Unionoida). The evolutionary biology of the bivalvia.

[ref-3] Bogan AE, Roe KJ (2008). Freshwater bivalve (Unioniformes) diversity, systematics, and evolution: status and future directions. Journal of the North American Benthological Society.

[ref-4] Boyer SL, Howe AA, Juergens NW, Hove MC (2011). A DNA-barcoding approach to identifying juvenile freshwater mussels (Bivalvia: Unionidae) recovered from naturally infested fishes. Journal of the North American Benthological Society.

[ref-5] Breton S, Beaupré HD, Stewart DT, Hoeh WR, Blier PU (2007). The unusual system of doubly uniparental inheritance of mtDNA: isn’t one enough?. Trends in Genetics.

[ref-6] Breton S, Beaupré HD, Stewart DT, Piontkivska H, Karmakar M, Bogan AE, Blier PU, Hoeh WR (2009). Comparative mitochondrial genomics of freshwater mussels (Bivalvia: Unionoida) with doubly uniparental inheritance of mtDNA: gender-specific open reading frames and putative origins of replication. Genetics.

[ref-7] Breton S, Ghiselli F, Passamonti M, Milani L, Stewart DT, Hoeh WR (2011a). Evidence for a fourteenth mtDNA-encoded protein in the female-transmitted mtDNA of marine mussels (Bivalvia: Mytilidae). PLOS ONE.

[ref-8] Breton S, Stewart DT, Hoeh WR (2010). Characterization of a mitochondrial ORF from the gender-associated mtDNAs of *Mytilus* spp. (Bivalvia: Mytilidae): identification of the missing ATPase 8 gene. Marine Genomics.

[ref-9] Breton S, Stewart DT, Shepardson S, Trdan RJ, Bogan AE, Chapman EG, Ruminas A, Piontkivska H, Hoeh WR (2011b). Novel protein genes in animal mtDNA: a new sex determination system in freshwater mussels (Bivalvia: Unionoida)?. Molecular Biology and Evolution.

[ref-10] Burdick RC, White MM (2007). Phylogeography of the Wabash pigtoe, *Fusconaia flava* (Rafinesque, 1820) (Bivalvia: Unionidae). Journal of Molluscan Studies.

[ref-11] Campbell DC, Johnson PD, Williams JD, Rindsberg AK, Serb JS, Small KK, Lydeard C (2008). Identification of ‘extinct’ freshwater mussel species using DNA barcoding. Molecular Ecology Resources.

[ref-12] Campbell D, Lydeard C (2012). The genera of Pleurobemini (Bivalvia: Unionidae: Ambleminae). American Malacological Bulletin.

[ref-13] Campbell DC, Serb JM, Buhay JE, Roe KJ, Minton RL, Lydeard (2005). Phylogeny of North American amblemines (Bivalvia, Unionoida): prodigious polyphyly proves pervasive across genera. Invertebrate Biology.

[ref-14] Chapman EG, Piontkivska H, Walker JM, Stewart DT, Curole JP, Hoeh WR (2008). Extreme primary and secondary protein structure variability in the chimeric male-transmitted cytochrome c oxidase subunit II protein in freshwater mussels: evidence for an elevated amino acid substitution rate in the face of domain-specific purifying selection. BMC Evolutionary Biology.

[ref-15] Curole JP, Kocher TD (2002). Ancient sex-specific extension of the cytochrome c oxidase II gene in bivalves and the fidelity of doubly-uniparental inheritance. Molecular Biology and Evolution.

[ref-16] Cyr F, Paquet A, Martel AL, Angers B (2007). Cryptic lineages and hybridization in freshwater mussels of the genus Pyganodon (Unionidae) in northeastern North America. Canadian Journal of Zoology.

[ref-17] Doucet-Beaupré H, Blier PU, Chapman EG, Piontkivska H, Sietman BE, Mulcrone RS, Hoeh WR (2012). Pyganodon (Bivalvia: Unionoida: Unionidae) phylogenetics: a male- and female-transmitted mitochondrial DNA perspective. Molecular Phylogenetics and Evolution.

[ref-18] Doucet-Beaupré H, Breton S, Chapman EG, Blier PU, Bogan AE, Stewart DT, Hoeh WR (2010). Mitochondrial phylogenomics of the Bivalvia (Mollusca): searching for the origin and mitogenomic correlates of doubly uniparental inheritance of mtDNA. BMC Evolutionary Biology.

[ref-19] Drummond AJ, Suchard MA, Xie D, Rambaut A (2012). Bayesian phylogenetics with BEAUti and the BEAST 1.7. Molecular Biology and Evolution.

[ref-20] Edgar RC (2004). MUSCLE: multiple sequence alignment with high accuracy and high throughput. Nucleic Acids Research.

[ref-21] Folmer O, Black M, Hoeh WR, Lutz R, Vrijenhoek RDNA (1994). Primers for the amplification of mitochondrial cytochrome c oxidase subunit I from diverse metazoan invertebrates. Molecular Marine Biology and Biotechnology.

[ref-22] Gershoni M, Templeton AR, Mishmar D (2009). Mitochondrial bioenergetics as a major motive force of speciation. Bioessays.

[ref-23] Gusman A, Lecomte S, Stewart DT, Passamonti M, Breton S (2016). Pursuing the quest for better understanding the taxonomic distribution of the system of doubly uniparental inheritance of mtDNA. PeerJ.

[ref-24] Harris JL, Hoeh WR, Christian AD, Walker JL, Farris JL, Johnson RL, Gordon ME (2004). Species limits and phylogeography of Lampsilinae (Bivalvia; Unionoida) in Arkansas with emphasis on species of *Lampsilis*. Little rock: final report to arkansas game and fish commission.

[ref-25] Harris JL, Posey WR, Davidson CL, Farris JL, Oetker SR, Stoeckel JN, Grump BG, Barnett MS, Martin HC, Seagraves JH, Matthews MW, Winterringer R, Osborne C, Christian AD, Wentz NJ (2010). Unionoida (Mollusca: Margaritiferidae, Unionidae) in Arkansas, third status review. Journal of the Arkansas Academy of Science.

[ref-26] Hebert PDN, Ratnasingham S, De Waard JR (2003). Barcoding animal life: cytochrome c oxidase subunit 1 divergences among closely related species. Proceedings of the Royal Society B: Biological Sciences.

[ref-27] Hoeh WR, Frazer KS, Naranjo-Garcia E, Trdan RJ (1995). A phylogenetic perspective on the evolution of simultaneous hermaphroditism in a freshwater mussel clade (Bivalvia: Unionidae: *Utterbackia*). Malacological Review.

[ref-28] Inoue K, Hayes DM, Harris JL, Christian AD (2013). Phylogenetic and morphometric analyses reveal ecophenotypic plasticity in freshwater mussels *Obovaria jacksoniana* and *Villosa arkansasensis* (Bivalvia: Unionidae). Ecology and Evolution.

[ref-29] IUCN (2015). The IUCN red list of threatened species. http://www.iucnredlist.org.

[ref-30] Kearse M, Moir R, Wilson A, Stones-Havas S, Cheung M, Sturrock S, Buxton S, Cooper A, Markowitz S, Duran C, Thierer T, Ashton B, Meintjes P, Drummond A (2012). Geneious basic: an integrated and extendable desktop software platform for the organization and analysis of sequence data. Bioinformatics.

[ref-31] Krebs RA (2004). Combining paternally and maternally inherited mitochondrial DNA for analysis of population structure in mussels. Molecular Ecology.

[ref-32] Krebs RA, Borden WC, Evans NM, Doerder FP (2013). Differences in population structure estimated within maternally- and paternally-inherited forms of mitochondria in *Lampsilis siliquoidea* (Bivalvia: Unionidae). Biological Journal of the Linnean Society London.

[ref-33] Kumar S, Stecher G, Tamura K (2016). MEGA7: molecular evolutionary genetics analysis version 7.0 for bigger datasets. Molecular Biology and Evolution.

[ref-34] Librado P, Rozas J (2009). DnaSP v5: a software for comprehensive analysis of DNA polymorphism data. Bioinformatics.

[ref-35] Lopes-Lima M, Sousa R, Geist J, Aldridge DC, Araujo R, Bergengren J, Bespalaya Y, Bodis E, Burlakova L, Van Damme D, Douda K, Froufe E, Georgiev D, Gumpinger C, Karatayev A, Kebapçi U, Killeen I, Lajtner J, Larsen BM, Lauceri R, Legakis A, Lois S, Lundberg S, Moorkens E, Motte G, Nagel KO, Ondina P, Outeiro A, Paunovic M, Prié V, Von Proschwitz T, Riccardi N, Rudsite M, Rudzitis M, Scheder C, Seddon M, Sereflisan H, Simic V, Sokolova S, Stoeckl K, Taskinen J, Teixeira A, Thielen F, Trichkova T, Varandas S, Vicentini H, Zajac K, Zajac T, Zogaris S (2017). Conservation status of freshwater mussels in Europe: state of the art and future challenges. Biological Reviews of the Cambridge Philosophical Society.

[ref-36] Lowe TM, Eddy SR (1997). tRNAscan-SE: a program for improved detection of transfer RNA genes in genomic sequence. Nucleic Acids Research.

[ref-37] Mathias PT, Hoffman JR, Wilson CC, Zanatta DT (2018). Signature of postglacial colonization on contemporary genetic structure and diversity of *Quadrula quadrula* (Bivalvia: Unionidae). Hydrobiologia.

[ref-38] McCartney MA, Bogan AE, Sommer KM, Wilbur AE (2016). Phylogenetic analysis of lake Waccamaw endemic freshwater mussel species. American Malacological Bulletin.

[ref-39] Milani L, Ghiselli F, Guerra D, Breton S, Passamonti M (2013). A comparative analysis of mitochondrial ORFans: new clues on their origin and role in species with doubly uniparental inheritance of mitochondria. Genome Biology and Evolution.

[ref-40] Mioduchowska M, Kaczmarczyk A, Zając K, Zając T, Sell J (2016). Gender-associated mitochondrial DNA heteroplasmy in somatic tissues of the endangered freshwater mussel Unio crassus (Bivalvia: Unionidae): implications for sex identification and phylogeographical studies. Journal of Experimental Zoology A Ecological and Inegrative Physiology.

[ref-41] Mitchell A, Guerra D, Stewart DT, Breton S (2016). In silico analyses of mitochondrial ORFans in freshwater mussels (Bivalvia: Unionoida) provide a framework for future studies of their origin and function. BMC Genomics.

[ref-42] Mulvey M, Lydeard C, Pyer DL, Hicks KM, Brim-Box J, Williams JD, Butler RS (1997). Conservation genetics of North American freshwater mussels *Amblema* and *Megalonaias*. Conservation Biology.

[ref-43] Palumbi S, Martin A, Romano WO, McMillan L, Stice L, Grabowski G (1991). The simple fools guide to PCR, version II.

[ref-44] Pfeiffer JM, Johnson NA, Randklev CR, Howells RG, Williams JD (2016). Generic reclassification and species boundaries in the rediscovered freshwater mussel ‘*Quadrula’* mitchelli (Simpson in Dall, 1896). Conservation Genetics.

[ref-45] Qvarnström A, Bailey RI (2009). Speciation through evolution of sex-linked genes. Heredity.

[ref-46] R Core Team (2015). https://www.r-project.org.

[ref-47] Rambaut A, Drummond A (2002). http://beast.community/version_history.html.

[ref-48] Rastogi P, Misener M, Krawetz S (1999). MacVector. Bioinformatics methods and protocols: methods in molecular biology.

[ref-49] Regnier C, Fontaine B, Bouchet P (2009). Not knowing, not recording, not listing: numerous unnoticed mollusk extinctions. Conservation Biology.

[ref-50] Roe KJ, Hartfield PD, Lydeard C (2001). Phylogeographic analysis of the threatened and endangered superconglutinate-producing mussels of the genus Lampsilis (Bivalvia: Unionidae). Molecular Ecology.

[ref-51] Serb JM, Lydeard C (2003). Complete mtDNA sequence of the North American freshwater mussel, *Lampsilis ornata* (Unionidae): an examination of the evolution and phylogenetic utility of mitochondrial genome organization in Bivalvia (Mollusca). Molecular Biology and Evolution.

[ref-52] Shearer TL, Coffroth MA (2008). DNA BARCODING: barcoding corals: limited by interspecific divergence, not intraspecific variation. Molecular Ecology Resources.

[ref-53] Sommer K (2007). Genetic identification and phylogenetics of Lake Waccamaw endemic freshwater mussel species. M.S. thesis.

[ref-54] Walker JM, Curole JP, Wade DE, Hoeh WR (2006). Taxonomic distribution and phylogenetic utility of gender-associated mitochondrial genomes in the Unionoida (Bivalvia). Malacologia.

[ref-55] Wares JP (2014). Mitochondrial cytochrome b sequence data are not an improvement for species identification in scleractinian corals. PeerJ.

[ref-56] Williams JD, Bogan AE, Butler RS, Cummings KS, Garner JT, Harris JL, Johnson NA, Watters GT (2017). A revised list of the freshwater mussels (Mollusca: Bivalvia: Unionida) of the United States and Canada. Freshwater Mollusk Biology and Conservation.

