# Supplementary figures and images for: Evaluating the utility of the female-specific mitochondrial f-orf gene for population genetic, phylogeographic and systematic studies in freshwater mussels (Bivalvia: Unionida)
Source: PeerJ. 2018 Jun 13;6:e5007. doi: 10.7717/peerj.5007 (PMC6004104; doi:10.7717/peerj.5007)

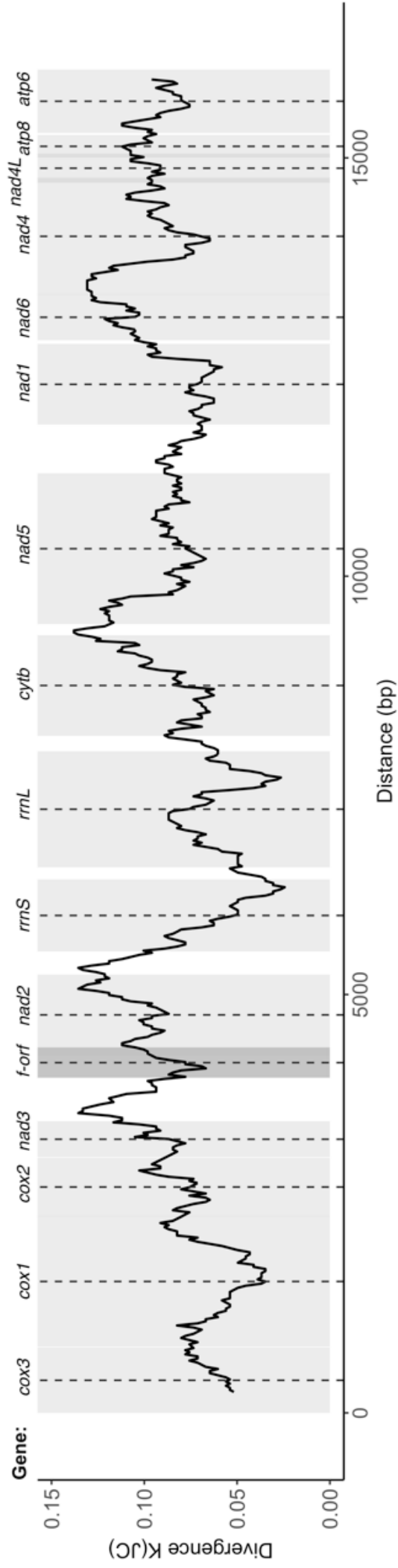

Supplement: Figure S1 — Analysis was conducted using a window size of 500 bp and 25 bp jumps. JC, Jukes Cantor correction. Light grey blocks represent protein-coding OXPHOS and ribosomal RNA gene regions, while the novel f-orf gene is highlighted dark grey. Dashed lines indicate the midpoint of genes. [file peerj-06-5007-s001.pdf]

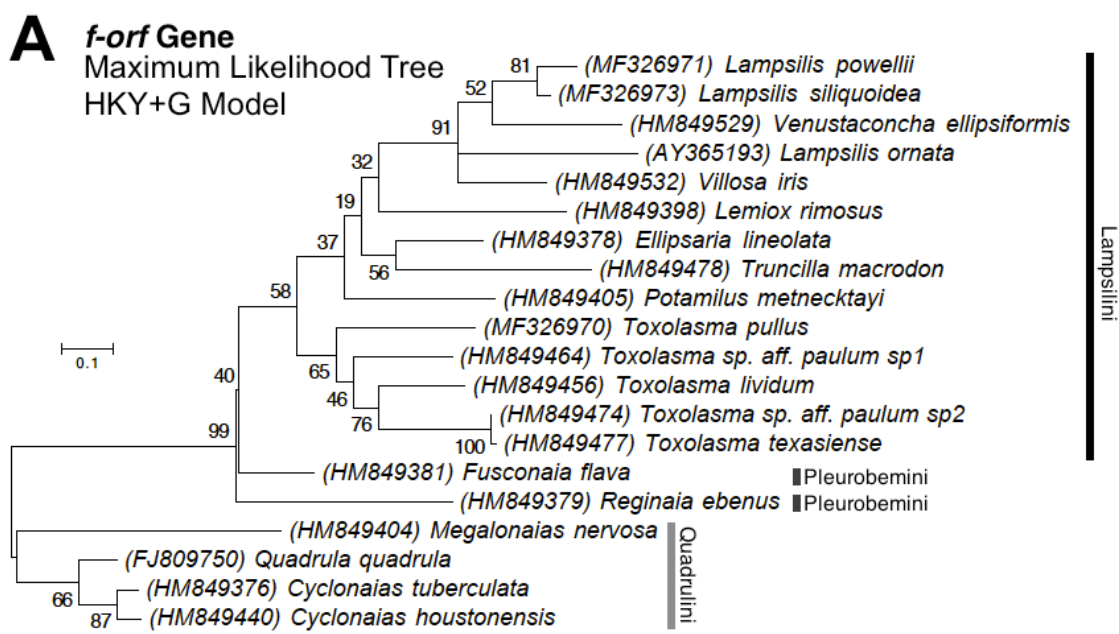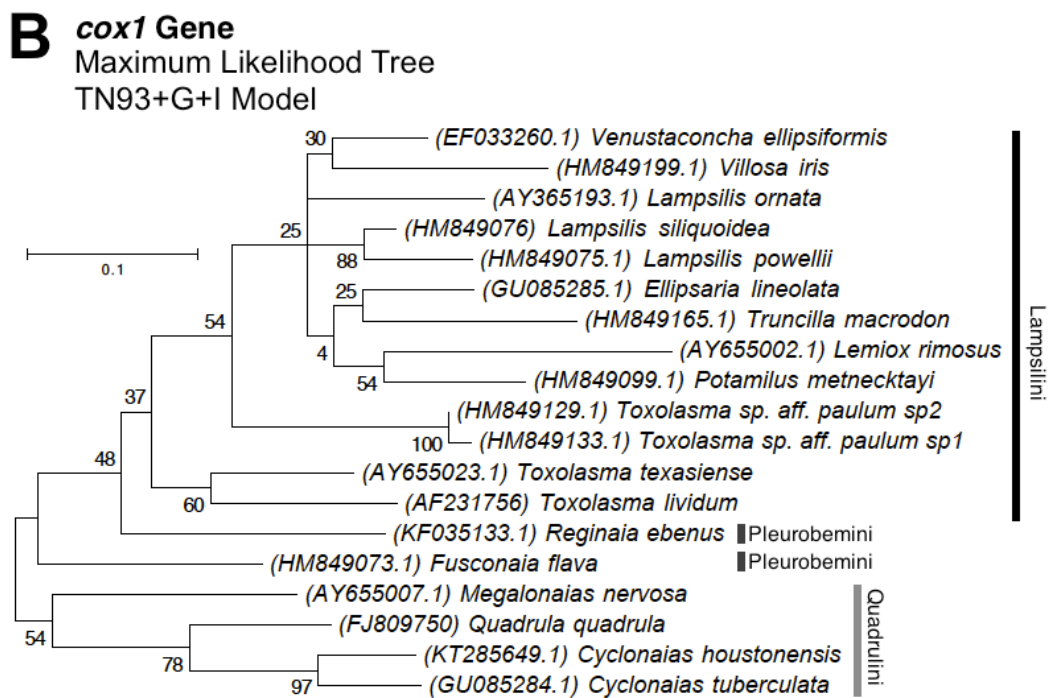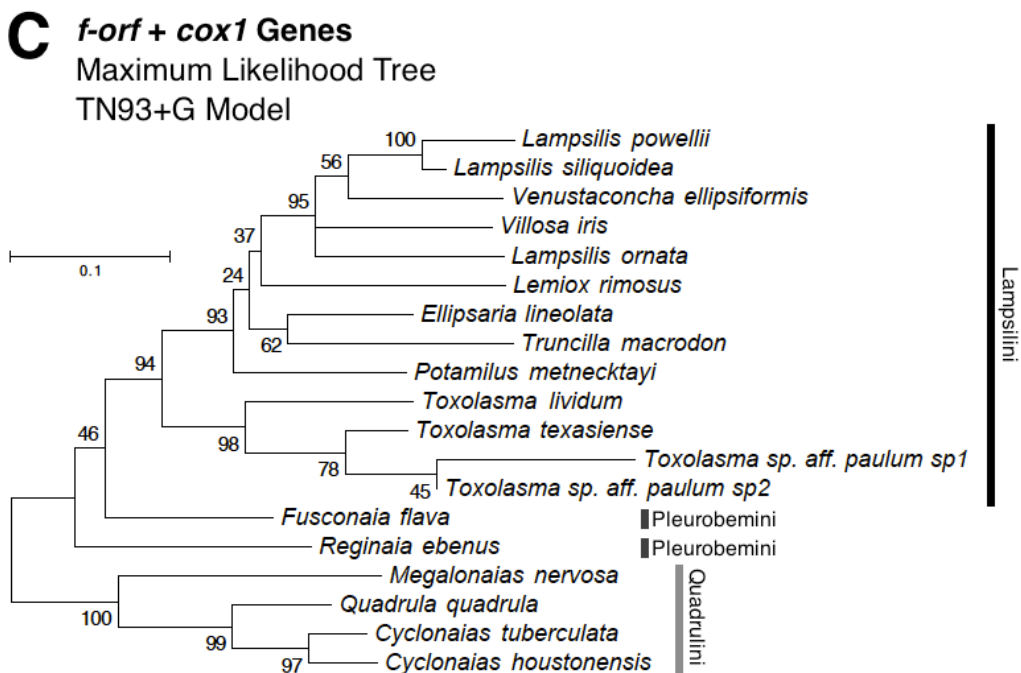

Supplement: Figure S2 — Samples/sequences used are described in Table 2. Substitution models selected (Hasegawa-Kishino-Yano (HKY) +G for f-orf, Tamura-Nei (TN93) +G +I for cox1, and TN93 +G for f-orf +cox1) were based on running alignments through MEGA’s model selection analysis. The best models for individual gene alignments had the lowest BIC scores for each gene. The top most similar model between the two individual model tests was chosen as the best model for the concatenated gene tree (again based on lowest BIC scores). Bootstrap percentage values based on 500 replicates are shown to the left of nodes. [file peerj-06-5007-s002.pdf]
